# Assessing the impact of discordant antibiotic treatment on adverse outcomes in community-onset UTI: a retrospective cohort study

**DOI:** 10.1093/jac/dkad357

**Published:** 2023-11-17

**Authors:** Anna Aryee, Patrick Rockenschaub, John Robson, Zaheer Ahmed, Caoimhe Nic Fhogartaigh, David Ball, Andrew Hayward, Laura Shallcross

**Affiliations:** Institute of Health Informatics, University College London, 222 Euston Road, London NW1 2DA, UK; Institute of Health Informatics, University College London, 222 Euston Road, London NW1 2DA, UK; Clinical Effectiveness Group, Queen Mary University of London, Yvonne Carter Building, 58 Turner Street, London E1 2AB, UK; Clinical Effectiveness Group, Queen Mary University of London, Yvonne Carter Building, 58 Turner Street, London E1 2AB, UK; Department of Microbiology, Barts Health NHS Trust, Pathology and Pharmacy Building, 80 Neward Street, London E1 2ES, UK; Department of Microbiology, Barts Health NHS Trust, Pathology and Pharmacy Building, 80 Neward Street, London E1 2ES, UK; Institute of Epidemiology and Health Care, University College London, 1-19 Torrington Place, London WC1E 7HB, UK; Institute of Health Informatics, University College London, 222 Euston Road, London NW1 2DA, UK

## Abstract

**Objectives:**

To investigate the risk of adverse outcomes following discordant antibiotic treatment (urinary organism resistant) for culture-confirmed community-onset lower urinary tract infection (UTI).

**Methods:**

Cohort study using routinely collected linked primary care, secondary care and microbiology data from patients with culture-confirmed community-onset lower UTI (COLUTI). Antibiotic treatment within ±3 days was considered concordant if the urinary organism was sensitive and discordant if resistant.

The primary outcome was the proportion of patients experiencing urinary infection-related hospital admission (UHA) within 30 days. Secondary outcomes were the proportion of patients experiencing reconsultation within 30 days, and the odds of UHA and reconsultation following discordant treatment, adjusting for sex, age, risk factors for complicated UTI, previous antibiotic treatment, recurrent UTI and comorbidities.

**Results:**

A total of 11 963 UTI episodes in 8324 patients were included, and 1686 episodes (14.1%, 95% CI 13.5%–14.7%) were discordant. UHA occurred in 212/10 277 concordant episodes (2.1%, 95% CI 1.8%–2.4%) and 88/1686 discordant episodes (5.2%, 95% CI 4.2%–6.4%). Reconsultation occurred in 3961 concordant (38.5%, 95% CI 37.6%–39.5%) and 1472 discordant episodes (87.3%, 95% CI 85.6%–88.8%). Discordant treatment compared with concordant was associated with increased odds of UHA (adjusted OR 2.31, 95% CI 1.77–3.0, *P* < 0.001) and reconsultation (adjusted OR 11.25, 95% CI 9.66–13.11, *P* < 0.001) on multivariable analysis. Chronic kidney disease and diabetes mellitus were also independently associated with increased odds of UHA.

**Conclusions:**

One in seven COLUTI episodes in primary care were treated with discordant antibiotics. In higher risk patients requiring urine culture, empirical antibiotic choice optimization could meaningfully reduce adverse outcomes.

## Introduction

Urinary tract infections (UTIs) are one of the commonest indications for antibiotic prescriptions in primary care. Studies have found that up to 50% of uncomplicated UTIs are self-limiting, and that symptomatic treatment may be as effective and safe as antibiotics in low-risk patients.^1–4^ Given the scale of prescribing for UTI, the use of such antimicrobial stewardship strategies would greatly reduce antibiotic consumption and therefore rates of antimicrobial resistance (AMR), which routine surveillance shows is increasing in both urine and blood culture isolates.^5,6^

Given the rising rates of AMR, however, treatment failure may be more likely in the subset of patients where antibiotic treatment is warranted, with adverse outcomes including pyelonephritis, urinary sepsis and bacteraemia. Treatment failure may also lead to reconsultations, adding demand on primary care.

UK guidelines recommend only sending urine cultures in cases of complicated UTI, treatment failure or where AMR is suspected, meaning that the subset of patients who have a urine culture sent are already deemed at higher risk of adverse outcomes.^7^ The majority of studies examining the impact of AMR on clinical outcomes have been carried out in hospital settings, and have broadly shown increased morbidity and mortality.^8^ Prior studies of treatment-failure UTI using routinely collected data lack microbiology data.^9,10^ Similarly, mandatory surveillance data on *Escherichia coli* urine and blood culture isolates lack prescribing data. We do not, therefore, have an accurate estimate of the impact of treatment failure due to AMR on adverse outcomes in patients who are treated for culture-confirmed community-onset UTI in primary care.

We aimed to use a dataset of linked primary care, secondary care and microbiology data to investigate the effect of discordant antibiotic treatment (urinary organism resistant) on adverse outcomes (hospitalization and primary care reconsultation) in the 30 days following a treated episode of culture-confirmed community-onset lower UTI (COLUTI) in primary care.

## Patients and methods

### Study design

This was a retrospective cohort study using routinely collected primary care data linked to secondary care and microbiological data. Primary care electronic healthcare records from patients registered at General Practices in East London, in a database managed by the Clinical Effectiveness Group (CEG, part of Queen Mary University of London), was deterministically linked to Secondary Uses Services (SUS) data (secondary care data) from Barts Health. SUS data are managed by NHS Digital and is the single comprehensive repository for healthcare data in England. These linked primary and secondary care data were then linked to microbiology data (urine and blood cultures) from Barts Health. The research question and protocol were framed prior to data extraction. UTI consultations were identified retrospectively through positive urine cultures with relevant uropathogens; see Table S1, available as Supplementary data at *JAC* Online. We identified distinct episodes of UTI using a 30 day washout period, with the start of the episode considered the first consultation date. We examined antibiotic prescriptions within a treatment window of ±3 days of the consultation to determine concordance: treatment with an antibiotic to which the urinary organism cultured was sensitive was considered concordant, and treatment with an antibiotic to which it was resistant was considered discordant (see Supplementary data). Consultations outside the 30 day washout period were considered a new episode. Consultations outside the treatment window but within the washout period were considered reconsultations (Figure S1). Reconsultations were also identified using a modified version of the Read code (a coded thesaurus of clinical terms, which have been used in the NHS since 1985) lists used in similar studies.^11,12^ Because antibiotics used to treat UTI are frequently unlinked to a diagnostic code, we also used prescriptions for nitrofurantoin, trimethoprim (alone, not in combination with sulfamethoxazole), fosfomycin and pivmecillinam to identify reconsultations.^13^ Trimethoprim can be used to treat other infections, such as respiratory tract infections and skin and soft tissue infections, but such use is infrequent in UK primary care (personal communication with GP A. Jhass, Institute of Health Informatics, UCL).

### Setting

Approximately 100 GP surgeries across two Clinical Commissioning Groups (CCGs, clinically led statutory NHS bodies responsible for the planning and commissioning of local healthcare services) in East London participated in the study. The study period was 1 April 2012 to 30 March 2017. The population represented is more ethnically diverse and socioeconomically deprived than the UK average.^14^

### Ethics and registration

The NHS Health Research Authority (HRA) toolkit (http://www.hra-decisiontools.org.uk/ethics/) identified that Research Ethics Approval was not required for this study as all data were pseudonymized and presented in aggregate form. HRA approval was received on 25 January 2018 (IRAS project ID 22683626836; REC reference 18/HRA/0502).

### Participants

Patients ≥16 years of age with a positive urine culture during the study period were eligible for inclusion. The following exclusions were applied (see Supplementary data): no record of age, sex or Index of Multiple Deprivation (IMD) score; inpatient urine samples; Read code indicating upper UTI (pyelonephritis) within ±3 days (Table S2); admission to hospital on day of consultation; registered for <12 months before urine sample; <30 days follow-up data after sample; and hospital discharge ≤30 days prior.

Patients entered the cohort at the start of their first episode of UTI and left the cohort at the earliest of these three dates: death; change of practice; or end of the study period.

### Exposure

The main variable of interest was discordant (urinary organism resistant) versus concordant (urinary organism sensitive) antibiotic treatment.

### Outcomes

The primary outcome was the proportion of episodes resulting in urinary infection-related hospital admission (UHA) in each treatment group (concordant and discordant). UHA was defined as an indicative ICD code (Table S8), or a positive urine or blood culture with a relevant uropathogen within 2 days of admission (Table S1). The secondary outcomes were the proportion of episodes resulting in reconsultation in each treatment group, and the odds of UHA and reconsultation with discordant treatment, adjusting for demographic variables and comorbidity.

### Other variables

Other variables examined (as outlined in the Supplementary data) were: (i) demographic variables (age, sex, ethnicity, IMD score); (ii) risk factors for complicated UTI: structural abnormalities, chronic kidney disease (CKD), urinary catheter in the past 6 months; (iii) recurrent UTI; (iv) comorbidities: cancer, diabetes mellitus (DM), heart failure, hypertension, urinary incontinence (UI), faecal incontinence (FI), obesity; (v) antibiotic exposure in preceding 6 months; and (vi) season of the year.

### Microbiology

The standard operating procedure (SOP) for handling of urine culture samples remained constant during the study period (see Supplementary data).

### Data sources

We created the cohort using data from a primary care database of East London general practices, developed and managed by the CEG at Queen Mary University of London. Data fields included sex, month and year of birth, ethnicity, IMD score, date of registration, Read codes for consultations and comorbidities, and prescriptions. These data were deterministically linked to SUS (managed by NHS Digital) secondary care data from Barts Health. We then linked these data to microbiology data (urine and blood cultures) from Barts Health (see Supplementary data).

### Statistical methods

We used descriptive statistics to summarize the clinical and demographic characteristics of UTI episodes. We reported age as the median with IQR and as a categorical variable. We categorized comorbidities as binary variables (present or not). We categorized previous antibiotic treatment as 0, 1–2 courses or ≥3 courses in the previous 6 months. We categorized IMD score as quintiles from 1 (least deprived) to 5 (most deprived). We categorized ethnic group as white, black, Asian, ‘mixed & other’, and unknown.

We assessed differences between exposure groups using Wilcoxon rank-sum tests for continuous variables and χ^2^ tests for categorical variables. We estimated crude associations (ORs) between each included variable and the outcomes using generalized estimating equations (GEEs) with a logit link and an exchangeable correlation structure accounting for multiple UTI episodes per patient. We used Huber–White sandwich estimators to calculate 95% CIs. We fitted a final multivariable adjusted model, including all predictors with a *P* value of <0.1 in the univariable analysis in addition to age category, sex and discordant treatment, which were included *a priori*. We performed all data cleaning and analyses using the statistical software R version 3.6.1 for Windows. We fitted GEEs using geepack (version 1.2-1).

## Results

**Figure 1.**
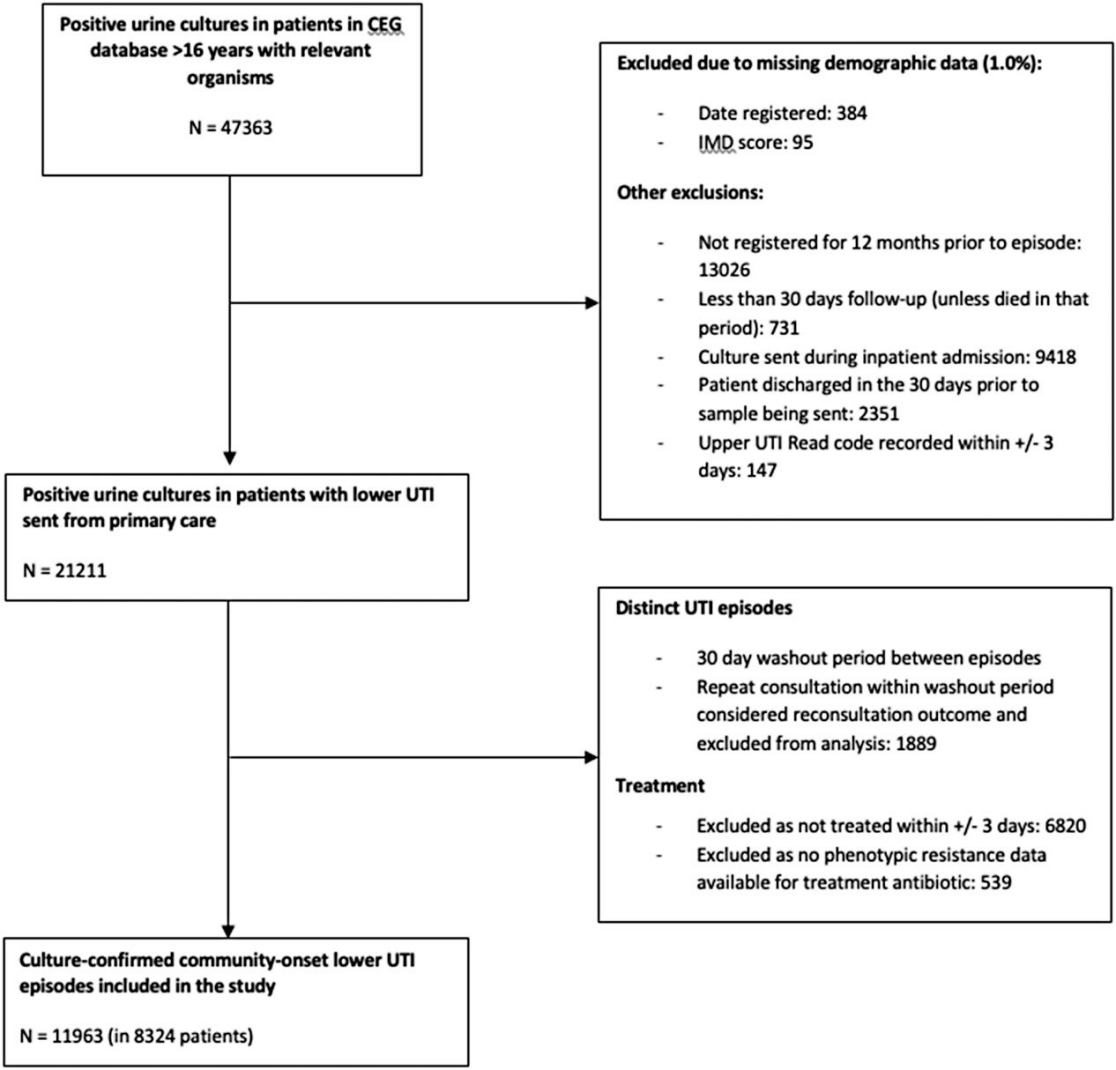
Flowchart of creation of cohort.

We included 11 963 episodes in 8324 patients, Figure 1. The commonest organism isolated was *Escherichia coli* (71.6%, 95% CI 70.8%–72.4%), followed by unspecified coliforms (15.5%, 95% CI 14.4%–15.7%), *Enterococcus* spp. (5.1%, 95% CI 4.7%–5.5%) and *Proteus* spp. (3.9%, 95% CI 3.6%–4.3%); Table 1. Of antibiotics prescribed within ±3 days of a positive urine culture, the commonest was trimethoprim (33.6%, 95% CI 32.8%–34.3%), followed by nitrofurantoin (23.7%, 95% CI 23.1%–24.4%), cefalexin (17.3%, 95% CI 16.7%–17.9%) and amoxicillin (15.7%, 95% CI 15.1%–16.3%); Table 2.

**Table 1.** Organisms identified on urine culture

Organism/species	Isolates, *n*	Proportion of total isolates, % (95% CI)
Total	12 016	100
*E. coli*	8600	71.6 (70.8–72.4)
Organism of the coliform group	1810	15.1 (14.4–15.7)
*Enterococcus* spp.	607	5.1 (4.7–5.5)
*Proteus* spp.	474	3.9 (3.6–4.3)
*Staphylococcus saprophyticus*	160	1.3 (1.1–1.6)
*Pseudomonas* spp.	97	0.8 (0.7–1)
Group B *Streptococcus*	81	0.7 (0.5–0.8)
*Klebsiella* spp.	64	0.5 (0.4–0.7)
*Enterobacter* spp.	49	0.4 (0.3–0.5)
*Acinetobacter* spp.	34	0.3 (0.2–0.4)
*Morganella morganii*	16	0.1 (0.1–0.2)
*Serratia* spp.	16	0.1 (0.1–0.2)
*Citrobacter* spp.	8	0.1 (0–0.1)

**Table 2.** Antibiotics prescribed within ±3 days of urine culture

Treatment antibiotic	Number of courses	Proportion of total courses, % (95% CI)
Total prescriptions	14 755	100
Trimethoprim	4951	33.6 (32.8–34.3)
Nitrofurantoin	3502	23.7 (23.1–24.4)
Cefalexin	2552	17.3 (16.7–17.9)
Amoxicillin	2319	15.7 (15.1–16.3)
Co-amoxiclav	1034	7 (6.6–7.4)
Ciprofloxacin	370	2.5 (2.3–2.8)
Septrin	9	0.1 (0–0.1)
Fosfomycin	8	0.1 (0–0.1)
Cefuroxime	5	<0.1
Cefadroxil	2	<0.1
Pivmecillinam	2	<0.1
Cefradine	1	<0.1

Discordant treatment was found in 1686/11 963 episodes (14.1%, 95% CI 13.5%–14.7%), 10 356/11 963 episodes occurred in female patients (86.6%, 95% CI 85.9%–87.2%) and the median age at time of the episode was 54 years (IQR 35–72); Table 3. Of all episodes, 39.6% (95% CI 38.8%–40.5%) occurred in patients of Asian ethnicity, and non-white patients were more likely to receive discordant treatment than white individuals; Table 3. Overall, UHA occurred in 300/11 963 episodes (2.5%, 95% CI 2.2%–2.8%).

**Table 3.** Concordant versus discordant episodes

Characteristic	Total, *n* (%)	Concordant, *n* (%)	Discordant, *n* (%)	*P* value^a^
Total	11 963 (100)	10 277 (85.9)	1686 (14.1)	<0.001
Male	1607 (13.4)	1363 (84.8)	244 (15.2)	<0.001
Female	10 356 (86.6)	8914 (86.1)	1442 (13.9)	<0.001
Age, years (continuous)	54 (IQR 35–72)			
Age, years (categorical)				
16–34	2849 (23.8)	2518 (88.4)	331 (11.6)	<0.001
35–54	3274 (27.4)	2865 (87.5)	409 (12.5)	<0.001
55–74	3368 (28.2)	2871 (85.2)	497 (14.8)	<0.001
75+	2472 (20.7)	2023 (81.8)	449 (18.2)	<0.001
IMD quintile				
1 (least deprived)	60 (0.5)	53 (88.3)	7 (11.7)	<0.001
2	295 (2.5)	256 (86.8)	39 (13.2)	<0.001
3	592 (4.9)	510 (86.1)	82 (13.9)	<0.001
4	4100 (34.3)	3477 (84.8)	623 (15.2)	<0.001
5 (most deprived)	6916 (57.8)	5981 (86.5)	935 (13.5)	<0.001
Ethnicity				
White	5796 (48.4)	5102 (88)	694 (12)	<0.001
Black	902 (7.5)	770 (85.4)	132 (14.6)	<0.001
Asian	4742 (39.6)	3954 (83.4)	788 (16.6)	<0.001
Mixed & other	296 (2.5)	249 (84.1)	47 (15.9)	<0.001
Unknown	227 (1.9)	202 (89)	25 (11)	<0.001
Risk factors for cUTI				
Structural abnormalities	1360 (11.4)	1165 (85.7)	195 (14.3)	<0.001
CKD	1609 (13.4)	1293 (80.4)	316 (19.6)	<0.001
Urinary catheter	470 (3.9)	389 (82.8)	81 (17.2)	<0.001
Antibiotic courses in last 6 months				
None	5175 (43.3)	4512 (87.2)	663 (12.8)	<0.001
1–2	4368 (36.5)	3775 (86.4)	593 (13.6)	<0.001
≥3	2420 (20.2)	1990 (82.2)	430 (17.8)	<0.001
Other risk factors				
Recurrent UTI	2072 (17.3)	1745 (84.2)	327 (15.8)	<0.001
UI	2214 (18.5)	1873 (84.6)	341 (15.4)	<0.001
FI	374 (3.1)	313 (83.7)	61 (16.3)	<0.001
Obesity	324 (2.7)	271 (83.6)	53 (16.4)	<0.001
Heart failure	394 (3.3)	300 (76.1)	94 (23.9)	<0.001
Hypertension	4338 (36.3)	3620 (83.4)	718 (16.6)	<0.001
Cancer	877 (7.3)	735 (83.8)	142 (16.2)	<0.001
DM	2800 (23.4)	2313 (82.6)	487 (17.4)	<0.001
Season				
Spring	2691 (22.5)	2277 (84.6)	414 (15.4)	<0.001
Summer	3020 (25.2)	2599 (86.1)	421 (13.9)	<0.001
Autumn	3157 (26.4)	2721 (86.2)	436 (13.8)	<0.001
Winter	3095 (25.9)	2680 (86.6)	415 (13.4)	<0.001
Outcomes				
UHA	300 (2.5)	212 (70.7)	88 (29.3)	<0.001
Reconsultation	5433 (45.4)	3961 (72.9)	1472 (27.1)	<0.001

cUTI, complicated UTI.

^a^χ^2^ test of proportions between concordant and discordant episodes.

UHA occurred in 212/10 277 concordant episodes (2.1%, 95% CI 1.8%–2.4%) and 88/1686 discordant episodes (5.2%, 95% CI 4.2%–6.4%); Table 4. Reconsultation occurred in 3961/10 277 concordant episodes (38.5%, 95% CI 37.6%–39.5%) and 1472/1686 discordant episodes (87.3%, 95% CI 85.6%–88.8%).

**Table 4.** Outcomes by concordant or discordant treatment

Outcome	Concordant, *n*	Concordant, % (95% CI)	Discordant, *n*	Discordant, % (95% CI)	*P* value^a^
Total episodes	10 277		1686		
UHA	212	2.1 (1.8–2.4)	88	5.2 (4.2–6.4)	<0.001
Reconsultation	3961	38.5 (37.6–39.5)	1472	87.3 (85.6–88.8)	<0.001

^a^χ^2^ test of proportion between concordant and discordant episodes.

Discordant antibiotic treatment was associated with increased odds of UHA compared with concordant treatment (adjusted OR 2.31, 95% CI 1.77–3.0, *P* < 0.001) on multivariable analysis adjusted for age, sex and all factors associated with the outcome on univariable analysis. Female sex was associated with reduced odds of UHA compared with male sex, but there was no association between UHA and age. Of other risks examined, CKD and DM were associated with increased odds of UHA; Table 5.

**Table 5.** Multivariable analysis of the odds of UHA

Characteristic	Adjusted OR (95% CI)	*P* value
Antibiotic treatment		
Concordant	1	
Discordant	2.31 (1.77–3)	<0.001
Male	1	
Female	0.58 (0.43–0.78)	<0.001
Age, years (categorical)		
16–34	1	
35–54	1.3 (0.87–1.94)	0.208
55–74	0.86 (0.54–1.36)	0.514
75+	1.31 (0.8–2.12)	0.282
Risk factors for cUTI		
Absence of risk factor	1	
Structural abnormalities	1.09 (0.77–1.54)	0.63
CKD	1.51 (1.07–2.13)	0.018
Urinary catheter	1.21 (0.73–2.01)	0.455
Antibiotic courses in last 6 months		
0	1	
1–2	0.98 (0.75–1.29)	0.885
≥3	1.06 (0.77–1.46)	0.732
Other risk factors		
Absence of risk factor	1	
Recurrent UTI	1.16 (0.84–1.61)	0.364
FI	1.13 (0.62–2.07)	0.694
Heart failure	1.41 (0.86–2.3)	0.175
Hypertension	1.23 (0.89–1.69)	0.213
DM	1.68 (1.26–2.23)	<0.001

cUTI, complicated UTI.

Discordant treatment was also associated with increased odds of reconsultation on multivariable analysis adjusted for age, sex and all variables associated with the outcome on univariable analysis (adjusted OR 11.25, 95% CI 9.66–13.11, *P* < 0.001); Table 6. Women had reduced odds of reconsultation compared with men, and individuals aged 55–74 years had increased odds of reconsultation compared with those aged 16–34 years.

**Table 6.** Multivariable analysis of odds of reconsultation

Characteristic	Adjusted OR (95% CI)	*P* value
Antibiotic treatment		
Concordant	1	
Discordant	11.25 (9.66–13.11)	<0.001
Male	1	
Female	0.76 (0.67–0.86)	<0.001
Age, years (categorical)		
16–34	1	
35–54	1.08 (0.96–1.21)	0.205
55–74	1.3 (1.13–1.49)	<0.001
75+	1.17 (0.99–1.38)	0.067
IMD quintile		
1 (least deprived)	1	
2	0.49 (0.23–1.04)	0.063
3	0.4 (0.19–0.82)	0.012
4	0.42 (0.21–0.85)	0.016
5 (most deprived)	0.45 (0.22–0.91)	0.026
Ethnicity		
White	1	
Black	0.85 (0.72–1)	0.045
Asian	1.03 (0.94–1.13)	0.558
Mixed & other	0.98 (0.75–1.27)	0.853
Unknown	0.82 (0.61–1.10)	0.189
Risk factors for cUTI		
Absence of risk factor	1	
Structural abnormalities	1.38 (1.21–1.58)	<0.001
CKD	1 (0.87–1.15)	0.955
Urinary catheter	1.14 (0.9–1.44)	0.282
Antibiotic courses in last 6 months		
0	1	
1–2	1.14 (1.04–1.24)	0.005
≥3	1.94 (1.72–2.19)	<0.001
Other risk factors		
Absence of risk factor	1	
Recurrent UTI	1.49 (1.32–1.68)	<0.001
UI	1.11 (0.99–1.25)	0.07
FI	1.1 (0.85–1.41)	0.482
Heart failure	1.0 (0.85–1.41)	0.99
Hypertension	1.13 (1.01–1.26)	0.038
Cancer	0.98 (0.83–1.16)	0.813
DM	0.95 (0.85–1.07)	0.382

cUTI, complicated UTI.

Of other risk factors examined, structural abnormalities, recurrent UTI and antibiotics in the preceding 6 months and hypertension were associated with increased odds of reconsultation compared with no history of these risk factors; Table 6. The number needed to treat (NNT) for UHA was 33 for 16–34 years, 27 for 34–54 years, 77 for 55–75 years and 26 for 75+ years. The NNT for reconsultation was 2 for 16–34 and 24–55 years, and 3 for 55–74 and 75+ years.

## Discussion

### Summary of findings

In this study of 11 963 culture-confirmed community-onset UTI episodes in 8325 patients, one in seven UTI episodes had discordant treatment, and this was associated with increased odds of both UHA and reconsultation. Women had reduced odds of UHA and reconsultation compared with men, and CKD and DM were both risk factors for UHA, irrespective of discordant treatment. Risk factors for reconsultation differed from risk factors for UHA and included structural abnormalities, recurrent UTI, prior antibiotics and hypertension.

### Comparison with other literature

Previous studies have lacked microbiology data and therefore used represcription of antibiotics as a proxy for treatment failure in COLUTI.^9,10,15–17^ A prospective cohort study of 497 women with COLUTI treated with trimethoprim found those with trimethoprim-resistant isolates had longer median time to symptom resolution (7 versus 4 days), more frequent reconsultation (36% versus 4% in the first week) and higher proportions of bacteriuria at 1 month (42% versus 20%).^18^ This study included relatively young women (median age 39 years, IQR 24–53) and did not examine hospitalization or resistance to other antibiotics.

The antibiotics prescribed within the treatment window of consultations were in keeping with UK primary care guidance on prescribing for UTI, with nitrofurantoin (if no renal impairment) and trimethoprim recommended as first-line agents for men and non-pregnant women aged ≥16 years. In pregnant women, nitrofurantoin is recommended as first-line agent (if no renal impairment and avoiding term), with amoxicillin and cefalexin recommended as second-line agents. Of note, prescribing guidance was changed in 2014 in response to changing AMR patterns to recommend nitrofurantoin as first-line agent, with trimethoprim only to be used if the risk of resistance was considered to be low.^19^ We observed a reduction in trimethoprim and increase in nitrofurantoin prescriptions during the study period (data not shown), in line with this change.

Women accounted for the majority of episodes, but had reduced odds of UHA compared with men. This concords with national surveillance on *E. coli* bacteraemia, showing higher rates in men than women, particularly in older patients. Large scale studies have previously found an increasing risk of sepsis with increasing age, but these studies have not been able to adjust for discordant treatment.^20^ We found that patients aged <55 years were more likely to receive concordant treatment, but that patients aged 75+ years were more likely to receive discordant treatment. This concords with sentinel surveillance of urine cultures, showing that the proportion of resistant isolates is higher in older age groups for both men and women.^21^

A significant proportion (45.4%, 95% CI 44.5%–46.3%) of patients had a reconsultation in the 30 days following their episode, and discordant treatment was associated with increased odds of reconsultation. The higher rate of reconsultation in those with discordant treatment is likely to be explained, at least in part, by patients being recalled to optimize their antibiotic therapy, emphasizing the additional costs associated with treating drug-resistant infections. For patients with renal impairment in whom nitrofurantoin is contraindicated, alternative antibiotic choices may have led to more discordant prescribing or use of antibiotics with a higher resistance profile. However, 38.5% of concordant episodes also resulted in a reconsultation, suggesting that treatment failure due to AMR is not the only reason for patients to reconsult. Given the high prevalence of comorbidity in this population, it is probable that a proportion of these reconsultations were for conditions unrelated to UTI.

We found that CKD was associated with increased odds of UHA. This is in keeping with the findings of a retrospective cohort study using linked health record data from 795 484 patients aged ≥65 years from 393 general practices in England between 2010 and 2016. Compared with an estimated glomerular filtration rate (eGFR) of >60 mL/min/1.73 m^2^, patients with an eGFR of <15 mL/min/1.73 m^2^ had greater odds of hospitalization for UTI (adjusted OR 1.68, 95% CI 1.01–2.82, *P* < 0.001) and sepsis.^22^ DM, for which prevalence was high in this cohort, was also associated with increased odds of UHA, in keeping with other studies that have found diabetic patients to be at risk of infection-related adverse outcomes following UTI.^23–28^

### Strengths and limitations

Our cohort combined prescribing and microbiology data, allowing investigation of the effect of discordant antibiotic treatment on adverse outcomes in patients treated for culture-confirmed COLUTI in a way not previously done.

Limitations to our study include those common to studies using routinely collected data, with data collected in short consultations focused on clinical care. Because primary care guidelines suggest only sending urine cultures in cases of complicated UTI, treatment failure or where AMR is suspected, our cohort is not representative of the overall primary care population with COLUTI, but may be of relevance to higher-risk patients. We also saw higher levels of resistance compared with national surveillance data on community urine samples, with resistance to trimethoprim seen in 40.1% of *E. coli* isolates (data not shown) compared with 34.0% from 2017 surveillance.^29^ However, there was no increasing trend in the proportion of discordant episodes during the study period, which varied between 6.0% to 20.8% per month (data not shown).

Our cohort did not include data on attendances or prescriptions from urgent care centres (UCCs) or A&E (not resulting in admission). We may therefore have underestimated the number of reconsultations. We may also have misclassified episodes as discordant if they subsequently received concordant treatment in these settings, tending to underestimate the adverse impact of discordant treatment. We were unable to include pregnancy as a variable as the Read code for UTI in pregnancy was rarely recorded, but acknowledge that our dataset will include a number of pregnant women who have had urine cultures sent as part of antenatal care.

Whilst we adjusted for a number of confounding variables, we acknowledge the risk of residual confounding. Our cohort represents an ethnically diverse, socioeconomically deprived urban population, and the results may only be relevant to other similar populations. We found that patients of non-white ethnicity were more likely to receive discordant treatment. This may be due to acquired resistance related to travel, which we did not have data on. This area warrants further research given the potential health inequality.

### Conclusions

To our knowledge, this is the first large-scale study using linked primary care, secondary care and microbiology data to investigate the relationship between discordant treatment for culture-confirmed COLUTI and adverse outcomes including hospitalization. One in seven patients received discordant treatment, and this was associated with increased risk of both UHA and reconsultation. The majority of studies examining AMR and clinical outcomes have been carried out in hospital settings, and have tended to show increased morbidity and mortality.^8^ Lack of microbiology data has to date precluded the use of routinely collected data to examine this issue in primary care, but our findings suggest that the same applies. We found that older patients had higher rates of resistant isolates, and that older age was not associated with increased odds of UHA on multivariable analysis adjusting for discordant treatment and comorbidity. This suggests that AMR may be a factor in the adverse outcomes seen in older age groups following COLUTI.

The aims of antibiotic stewardship programmes are to reduce antibiotic prescribing where it is not warranted, and to ensure appropriate and targeted therapy in cases where it is. Studies have found that a significant proportion of uncomplicated lower UTIs in women are self-limiting, with up to 50% of participants symptom free without antibiotic treatment at 7 days.^1,2^ Similarly, trials in young women have found that approximately 2/3 of participants recover with symptomatic treatment rather than antibiotics, although this was associated with a higher burden of symptoms and, in one study, more cases of pyelonephritis.^3,4^ Given the number of patients treated for UTI, reductions in antibiotic prescribing, where appropriate, will likely have an impact on reducing rates of AMR. However, in the subset of higher risk patients where antibiotic treatment is warranted, our findings suggest that there is scope for improvement in individual patient management, for example with risk stratification based on prior antibiotic exposure, local antibiograms and/or rapid diagnostics, which may be of particular benefit in older patients.^30^ The delay between sample collection and antibiotic susceptibility testing (AST) results following bacterial culture delays targeted antibiotic treatment for UTI. Urine dipstick tests have a poor positive predictive value and are not recommended for use in patients >65 years.^7,31^ Near-patient tests that report on AST are being developed.^32,33^ Further studies should examine the cost-effectiveness of such diagnostics in the subset of patients who are at higher risk of AMR, and higher-risk patient groups such as those with DM, CKD or advanced age. In patients aged 75+ years, the NNT to prevent one UHA was 26 and for reconsultation it was 3, suggesting this may be a particular group in which to target such studies.

## Supplementary Material

dkad357_Supplementary_Data

## Supplementary data

Figure S1 and Tables S1–S8 are available as Supplementary data at *JAC* Online.
