# Supplementary material for: Assessing the impact of discordant antibiotic treatment on adverse outcomes in community-onset UTI: a retrospective cohort study
Source: J Antimicrob Chemother. 2023 Nov 17;79(1):134–42. doi: 10.1093/jac/dkad357 (PMC10761259; doi:10.1093/jac/dkad357)
Supplement: dkad357_Supplementary_Data [file dkad357_supplementary_data.docx]

**Clinical Effectiveness Group (CEG) database**

The Clinical Effectiveness Group (CEG), part of Queen Mary University of London, uses primary care electronic healthcare records to obtain non-identifiable patient information in order to help plan and improve healthcare services. CEG collaborates with a number of partner organisations: City & Hackney, Newham and Tower Hamlets Clinical Commissioning Groups (CCGs) and local authority public health teams, Queen Mary University of London and University College London Partners researchers, the local General Practice provider Federations, Barts Health Trust and the Commissioning Support Unit.

The CEG database contains primary care data on patients registered at General Practices in the three CCGs City & Hackney, Newham and Tower Hamlets, and currently has a registered population of approximately 1.2 million individuals. As general practice serves as the first point of contact in the UK healthcare system, and the vast majority of the population is registered with a GP, this database provides a very complete population sample. The data from the CEG database is extracted centrally from the web-enabled record system Egton Medical Information Services (EMIS), which is used by all the contributing practices to record consultations and prescriptions and includes agreed data-entry templates that ensure data entry and coding is consistent. Prescriptions are recorded electronically, so this data is highly complete. Consultation data, which is recorded using Read codes (the standard clinical terminology used in general practice in the UK), may be less complete. A number of Read codes are related to the Quality and Outcomes Framework (QOF), which is a voluntary annual reward and incentive programme for all GP surgeries in England, and so data on these may be recorded more consistently.

The primary care data can be deterministically linked to Secondary Uses Services (SUS) data (secondary care data) from Barts Health. SUS data is managed by NHS Digital and is the single comprehensive repository for healthcare data in England. Microbiology data is not fed back from the associated laboratories in coded form, and is reliant on clinicians entering it manually. A novel element in this study is linking this primary and secondary care data to microbiology data from Barts Health in order to combine data on primary care consultations and prescribing, hospital admissions and microbiological outcomes.

General practices (as data controllers) opt into the database, but individual patients can opt out. CEG (as data processor) has the written consent of all practices in the study area to use pseudonymised patient data for audit and research for patient benefit. The researchers adhere to the data protection principles of the Data Protection Act 2018, and all data is managed according to UK NHS information governance requirements. The database has been used to facilitate a number of research projects aiming at improving the effective delivery of primary care in an ethnically diverse, inner city environment[1–3].

**Identification of UTI Consultations through Positive Urine Cultures**

COLUTI consultations were identified through positive urine cultures with relevant organisms, as shown in Table S1. Organisms included were mainly Gram-negative bacteria, with the exception of *Staphylococcus saprophyticus*, Group B streptococcus and enterococci. As we were interested in bacterial UTI, Candida species and other yeasts were also excluded. We excluded urine cultures sent during an inpatient admission, as well as those sent within +/- 3 days of a Read code for upper UTI, Table S2. The same list of organisms was used to identify relevant organisms isolated on blood culture in order to identify urinary-related hospital admissions, as described below.

**Definition of UTI episodes**

Urine cultures were ordered by patient identifier and date, and a 30-day washout period was used to identify new episodes, so that any consultations within that period were considered part of the same episode. The start of the episode was considered as the first urine culture sample date. Any urine culture sent outside of the 30 day washout period was considered a new episode. If a repeat urine culture, Read code for UTI or antibiotic used to treat UTI (see antibiotic treatment definition below) was recorded within the washout period of an episode, the episode was defined as having the reconsultation outcome. In this case the reconsultation urine culture was excluded from the analysis, Figure S1. Patients could contribute more than one UTI episode to the analysis.


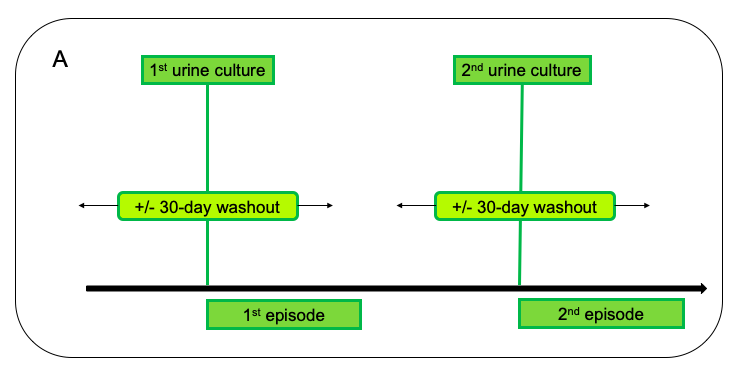


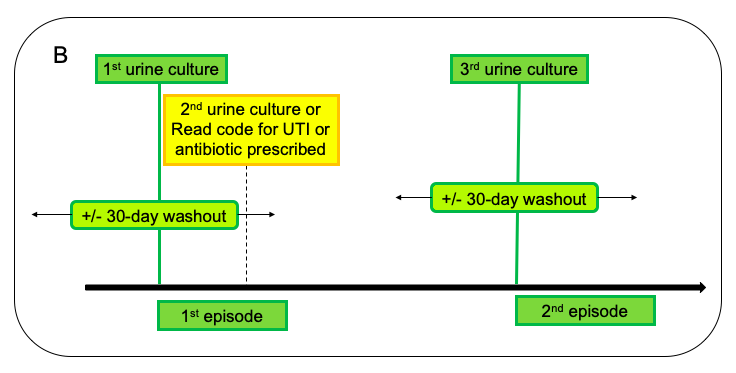


**Figure S1.** Definition of UTI episodes (adapted from Shallcross et al.)[5]

In both panels the 1^st^ urine culture represents the start of a new UTI episode (first episode). The 2^nd^ urine culture is classified as (A) a new episode (because it occurs outside the washout period of the previous culture); (B) a reconsultation because it occurs within the washout period of the previous culture. In panel B, the 1^st^ episode is given the outcome of reconsultation and the 2^nd^ urine culture is excluded from the analysis. A Read code for UTI or prescription of an antibiotic to treat UTI also results in a reconsultation outcome for the 1^st^ episode.

**Identification of UTI reconsultations through READ Codes**

We identified reconsultations for community-onset UTI (COLUTI) using a modified version of the Read code lists used in previous similar studies[4,5]. The list includes codes for suspected UTI, confirmed UTI, UTI symptoms and UTI tests that are indicative of a consultation for UTI, Table S3.

**Identification of UTI reconsultations through prescriptions**

A recent study of antibiotic prescriptions in primary care found that the first-line antibiotics used to treat UTI, nitrofurantoin and trimethoprim, were frequently unlinked to a diagnostic code (44.7% and 37.4% of prescriptions respectively)[6]. We therefore also identified COLUTI reconsultations through prescriptions of antibiotics that are primarily used to treat UTI in primary care – trimethoprim (alone, not in combination with sulfamethoxazole), nitrofurantoin, fosfomycin and pivmecillinam. Other antibiotics such as amoxicillin, cefalexin and co-amoxiclav were not used to identify reconsultations as these are frequently used to treat infections other than UTI. Trimethoprim can also be used to treat other infections, such as respiratory tract infections and skin and soft tissue infections, but such use is infrequent in primary care (personal communication with GP). Trimethoprim prescriptions were identified up to and including the equivalent of 200mg twice daily for 7 days, and nitrofurantoin up to and including the equivalent of 100mg twice daily for 7 days (the maximum duration for treatment of lower UTI). Courses longer than this were considered to represent antibiotic prophylaxis rather than treatment.

**Definition of discordance**

First line antibiotics tested for on urine cultures at Barts Health during the study period were:

Ampicillin (amoxicillin), cefalexin, gentamicin, ciprofloxacin, nitrofurantoin, trimethoprim, augmentin (co-amoxiclav), and cefpodoxime (also used as indicator antibiotic for ESBL-producing organisms).

Phenotypic antibiotic sensitivity data was reviewed for each episode. Treatment antibiotic was matched to phenotypic sensitivity with a treatment window of +/- 3 days. Treatment was considered concordant if the episode was treated with at least one antibiotic to which the organism was sensitive in the treatment window, and discordant if the episode was treated only with antibiotics to which the organism was resistant or intermediately resistant. In the case of multiple treatment antibiotics, only one of the antibiotics needed to be concordant for the episode to be considered concordant. Where more than one organism was isolated on the urine culture, phenotypic resistance was aggregated to the most resistant phenotype and treatment antibiotic was matched on this phenotype for the sample.

Where sensitivity data was not available the following rules were used:

- Sensitivity to co-amoxiclav was inferred from sensitivity to amoxicillin
- Sensitivity to levofloxacin was inferred from sensitivity to ciprofloxacin
- Sensitivity and resistance to other 1^st^ generation cephalosporins was inferred from sensitivity and resistance to cefalexin
- Sensitivity to 2^nd^ and 3^rd^ generation cephalosporins was inferred from sensitivity to cefalexin
- Resistance to 1^st^ generation cephalosporins was inferred from resistance to 2^nd^ and 3^rd^ generation cephalosporins
- Resistance to 2^nd^ generation cephalosporins was inferred from resistance to 3^rd^ generation cephalosporins[7].

Samples where there was no sensitivity data on the treatment antibiotic, and sensitivity could not be inferred from other phenotypic sensitivity results, were excluded.

**Definition of Recurrent UTI**

We identified recurrent UTI in 3 ways:

- A Read code for recurrent UTI (as shown in Table S4)
- A prescription for prophylactic nitrofurantoin or trimethoprim (defined as a dose of 100mg daily for each drug for a duration of greater than 28 days)
- 2 UTI consultations in 6 months, or 3 consultations in 12 months

A patient was considered to have a history of recurrent UTI if their UTI episode occurred in the 12 months following any of the above definitions, up to and including the date of their episode.

**Definition of Co-Morbidities**

A number of comorbidities have been shown to increased risk of UTI or increased risk of adverse outcomes. UTI in the presence of structural abnormalities of the renal tract or a functionally abnormal renal tract are by definition complicated infections, as are those in men and pregnant women. Indwelling urinary catheters are associated with increased risk of UTI, and catheter associated UTI (CAUTI) is one of the commonest healthcare-associated infections worldwide[8,9]. Diabetes mellitus (DM), urinary incontinence, cancer, heart failure and hypertension were identified as potential risk factors for community-onset *Escherichia coli* bacteraemia in a number of studies[10–13]. Further studies have found an association between obesity and UTI[14–16].

Pregnancy was identified through a Read code indicating an infection in pregnancy (L1668: Urinary tract infection complicating pregnancy), but as this Read code was only recorded on 16 occasions, these episodes were excluded and a variable for pregnancy was not included in the analysis. The comorbidities described above were identified through the Read codes shown in Table S5 as the first date they were recorded. Comorbidities were considered present at the time of the episode if the date they were recorded preceded the date of the UTI episode. Structural abnormalties of the renal tract were identified using the Read codes in Table S6.

In addition to Read codes, urinary incontinence, faecal incontinence and urinary catheters were identified through prescriptions for devices as shown Table S7. A catheter was considered present if the Read code for the episode indicated CAUTI, or if there was a Read code or device code for a catheter recorded in the 6 months preceding the UTI episode.

**Definition of Previous Antibiotic Use**

We identified all systemic antibiotic prescriptions recorded for patients in the cohort in the period from 7 days before to 6 months before each episode. Prescriptions issued on the same day were counted as the same treatment course, and further prescriptions on a later date were counted as separate treatment courses.

**Definition of Season**

Season of the year has been shown to be associated with UTI, with a peak in consultation incidence seen in autumn and increased incidence of *Escherichia coli* bacteraemia in summer [17–19]. We classified UTI episodes into seasons depending on the months in which they occurred:

Spring: March, April or May

Summer: June, July or August

Autumn: September, October or November

Winter: December, January or February

**Definition of Outcomes**

We defined urinary infection-related hospital admission (UHA) in the 30 days following a UTI episode using the ICD code list shown in Table S8 (adapted from Shallcross *et al.*) and included ICD codes for lower UTI, upper UTI, bloodstream infection or sepsis. An admission was considered UHA if the primary or secondary (in position 2) ICD code for that admission was included in this list, or if the patient had a positive urine or blood culture with a relevant organism (as per Table S2) within 2 days of admission. Multiple admissions in the 30 days following a consultation were aggregated and if any of them were considered UHA, the UTI episode was given the outcome UHA.

**Microbiology**

The SOP for handling of urine specimens at Barts Health was the same throughout the study period. Generally, only midstream urine samples, catheter specimens or suprapubic aspirated urine samples should be accepted for bacterial culture. Samples with a pure growth of ≥ 10^5^ organisms are generally considered significant, but in samples with large numbers of white blood cells a lower, or mixed colony count may be considered relevant. This relevance depends on the age and/or type of sample and the quality of the specimen, factors which are mainly ascertained by the presence or absence of epithelial cells.

The majority of urine specimens undergo automated microscopy using a Sysmex UF1000 analyser. Samples that are unsuitable for processing on the UF1000 include those described as “frank pus”, “gross blood”, those containing visible particulate matter or mucous, and those containing gross amounts of crystals or amorphous debris. These samples are processed manually.

The majority of samples are also cultured in an automated manner using a Biomerieux PREVI Isola, which identifies microscopy positive samples and samples them onto chromogenic UTI agar plates. Samples rejected by the PREVI but still requiring cultures are sampled manually onto chromogenic agar plates.

For general urines, phenotypic resistance testing is carried out for the following 1^st^ line antibiotics:

- Ampicillin
- Cefalexin
- Gentamicin
- Ciprofloxacin
- Nitrofurantoin
- Trimethoprim
- Augmentin (Co-amoxiclav)
- Cefpodoxime

2^nd^ line antibiotics are set up if there is sensitivity to <3 antibiotics on 1^st^ line, or if the isolate is resistant to cefpodoxime:

- Ertapenem
- Fosfomycin
- Amikacin
- Mecillinam
- Meropenem
- Temocillin
- Piperacillin/tazobactam

For general urines, if an enhanced inhibition zone is seen between the cefpodoxime and augmentin (co-amoxiclav) disc, known as the “keyhole” sign which is an indicator of ESBL production, the isolate is reported as ESBL+ and 2^nd^ line sensitivities are set up. If cefpodoxime is resistant and no keyhole is seen, further testing for ESBL and AmpC β-lactamase detection is set up as follows. 4 discs are used: cefotaxime 30ug; cefotaxime 30ug + clavulanic acid; cefotaxime 30ug and cloxacillin; cefotaxime 30ug, clavulanic acid and cloxacillin. The zones diameters are read and entered into an algorithm on the Winpath system which determines the presence of ESBL, AmpC β-lactamase or both, using pre-defined rules. Intermediate or resistance to meropenem, ertapenem, piperacillin/tazobactam or temocillin, or a combination of the above, is considered a presumptive carbapenem-resistant organism. These organisms are reported as such and the isolate sent to the PHE reference laboratory for further testing.

**Data linkage**

Pseudonymisation of data was carried out using the OpenPseudonymiser (OpenP) software, an open source standalone windows desktop application developed by the University of Nottingham[20]. OpenP allows creation of a pseudo ID (called the “digest”) from the NHS number, allowing record linkage to be undertaken without disclosure of patient identifiable data. In order to link data from the CEG database to microbiology data, a data scientist at CEG (Marian Priebe) used OpenP to create a pseudo ID from the NHS number of the included participants in the CEG database. The same software was used to create a pseudo ID from the patient NHS number of all patients with a positive urine or blood culture with a relevant organism at Barts Health during the study, by an NHS Senior Biomedical Scientist (David Ball). This microbiology data was saved onto an encrypted memory stick with the pseudo ID, but without the NHS number or any other identifiable data, and then deterministically linked to the database using the pseudo ID. This linkage was carried out under guidance by the CEG led for Information Governance, Dr Kambiz Boomla.

The data was uploaded in CSV files to the UCL Data Safe Haven (DSH), a service which provides a secure solution for storage, handling and analysis of identifiable data, on 18/12/18. The DSH has been certified to the ISO27001 information security standard and conforms to NHS Digital’s Information Governance Toolkit. A schematic of the data included in the study dataset is shown in Figure S2.

**Figure S2. Schematic of data included in cohort**

**References**

1. Schofield P, Das-Munshi J, Mathur R, Congdon P, Hull S. Does depression diagnosis and antidepressant prescribing vary by location? Analysis of ethnic density associations using a large primary-care dataset. Psychological Medicine. 2016;46(6):1321–9.

2. Homer K, Boomla K, Hull S, Dostal I, Mathur R, Robson J. Statin prescribing for primary prevention of cardiovascular disease: A cross-sectional, observational study. British Journal of General Practice. 2015;65(637):e538–44.

3. Robson J, Dostal I, Madurasinghe V, Sheikh A, Hull S, Boomla K, et al. The NHS Health Check programme: Implementation in east London 2009-2011. BMJ Open. 2015;5(4).

4. Gharbi M, Drysdale JH, Lishman H, Goudie R, Molokhia M, Johnson AP, et al. Antibiotic management of urinary tract infection in elderly patients in primary care and its association with bloodstream infections and all cause mortality: Population based cohort study. BMJ (Online). 2019;364.

5. Shallcross L, Rockenschaub P, Blackburn R, Nazareth I, Freemantle N, Hayward A. Antibiotic prescribing for lower UTI in elderly patients in primary care and risk of bloodstream infection: A cohort study using electronic health records in England. PLoS Medicine. 2020;17(9).

6. Dolk FCK, Pouwels KB, Smith DRM, Robotham J V., Smieszek T. Antibiotics in primary care in England: Which antibiotics are prescribed and for which conditions? Journal of Antimicrobial Chemotherapy. 2018;73:ii2–10.

7. Livermore DM, Winstanley TG, Shannon KP. Interpretative reading: Recognizing the unusual and inferring resistance mechanisms from resistance phenotypes. Journal of Antimicrobial Chemotherapy. 2001;48(SUPPL. 1):87–102.

8. Letica-Kriegel AS, Salmasian H, Vawdrey DK, Youngerman BE, Green RA, Furuya EY, et al. Identifying the risk factors for catheter-associated urinary tract infections: A large cross-sectional study of six hospitals. BMJ Open. 2019;9(2).

9. World Health Organisation. Report on the Burden of Endemic Health Care-Associated Infection Worldwide. WHO Library Cataloguing-in-Publication Data. 2011.

10. Jackson LA, Benson P, Neuzil KM, Grandjean M, Marino JL. Burden of community-onset Escherichia coli bacteremia in seniors. The Journal of infectious diseases. 2005;191(9):1523–9.

11. Thomsen RW, Hundborg HH, Lervang H-H, Johnsen SP, Schonheyder HC, Sorensen HT. Diabetes mellitus as a risk and prognostic factor for community-acquired bacteremia due to enterobacteria: a 10-year, population-based study among adults. Clinical infectious diseases : an official publication of the Infectious Diseases Society of America. 2005;40(4):628–31.

12. Park SH, Choi S-M, Lee D-G, Kim J, Choi J-H, Kim S-H, et al. Emergence of extended-spectrum beta-lactamase-producing escherichia coli as a cause of community-onset bacteremia in South Korea: risk factors and clinical outcomes. Microbial drug resistance (Larchmont, NY). 2011;17(4):537–44.

13. Kang C-I, Chung DR, Ko KS, Peck KR, Song J-H, (KONSID) KN for S of ID. Clinical predictors of Enterobacter bacteremia among patients admitted to the ED. The American journal of emergency medicine. 2012;30(1):165–9.

14. Alhabeeb H, Baradwan S, Kord-Varkaneh H, Tan SC, Low TY, Alomar O, et al. Association between body mass index and urinary tract infection: a systematic review and meta-analysis of observational cohort studies. Eating and Weight Disorders. 2021;26(7):2117–25.

15. Saliba W, Barnett-Griness O, Rennert G. The association between obesity and urinary tract infection. European Journal of Internal Medicine. 2013;24(2):127–31.

16. Semins MJ, Shore AD, Makary MA, Weiner J, Matlaga BR. The impact of obesity on urinary tract infection risk. Urology. 2012;79(2):266–9.

17. Al-Hasan MN, Lahr BD, Eckel-Passow JE, Baddour LM. Seasonal variation in Escherichia coli bloodstream infection: A population-based study. Clinical Microbiology and Infection. 2009;15(10):947–50.

18. Deeny SR, van Kleef E, Bou-Antoun S, Hope RJ, Robotham J V. Seasonal changes in the incidence of Escherichia coli bloodstream infection: variation with region and place of onset. Clinical microbiology and infection : the official publication of the European Society of Clinical Microbiology and Infectious Diseases. 2015;21(10):924–9.

19. Rosello A, Pouwels KB, Domenech De Cellès M, Van Kleef E, Hayward AC, Hopkins S, et al. Seasonality of urinary tract infections in the United Kingdom in different age groups: Longitudinal analysis of The Health Improvement Network (THIN). Epidemiology and Infection. 2018;146(1):37–45.

20. Nottingham U of. OpenPseudonymiser [Internet]. [cited 2021 Feb 1]. Available from: https://www.openpseudonymiser.org/Default.aspx

**Table S1. Organisms, species and descriptions included in urine cultures**

| Organism / species / description |
| --- |
| *Acinetobacter* spp |
| *Citrobacter* spp |
| *Enterobacter* spp |
| *Enterococcus* spp |
| *Escherichia coli* |
| Gram-negative rods |
| Group B streptococcus |
| *Klebsiella* spp |
| *Morganella* spp |
| Organism of the coliform group |
| *Proteus* spp |
| *Pseudomonas* spp |
| *Serratia* spp |
| *Staphylococcus saprophyticus* |

**Table S2. Read codes for upper UTI used to exclude consultations if recorded within +/- 3 days of urine culture**

| Read code | Description |
| --- | --- |
| K10000 | Chronic pyelonephritis |
| K100000 | Chronic pyelonephritis without medullary necrosis |
| K100100 | Chronic pyelonephritis with medullary necrosis |
| K100400 | Nonobstructive reflux-associated chronic pyelonephritis |
| K100500 | Chronic obstructive pyelonephritis |
| K100600 | Calculous pyelonephritis |
| K100z00 | Chronic pyelonephritis NOS |
| K10100 | Acute pyelonephritis |
| K101000 | Acute pyelonephritis without medullary necrosis |
| K101z00 | Acute pyelonephritis NOS |
| K10400 | Xanthogranulomatous pyelonephritis |
| K10y00 | Pyelonephritis and pyonephrosis unspecified |
| K10y000 | Pyelonephritis unspecified |
| K10y300 | Pyelonephritis in diseases EC |
| K10yz00 | Unspecified pyelonephritis NOS |
| K100200 | Chronic pyelitis |
| K10y400 | Pyelitis in diseases EC |
| K101200 | Acute pyelitis |
| K10y100 | Pyelitis unspecified |
| K102000 | Renal abscess |
| K10200 | Renal and perinephric abscess |
| K102100 | Perinephric abscess |
| K102z00 | Renal and perinephric abscess NOS |
| K10..00 | Infections of kidney |
| K10z00 | Infection of kidney NOS |
| K1011 | Renal infections |
| K1000 | Infections of kidney |
| K10z00 | Infection of kidney NOS |
| K21300 | Prostatocystitis |
| K10y200 | Pyonephrosis unspecified |
| K10500 | Chronic infective interstitial nephritis |

**Table S3. Read Codes used to identify UTI reconsultations**

| Read code | Description |
| --- | --- |
| 1AG..00 | Recurrent urinary tract infections |
| 1J4..00 | Suspected UTI |
| K15..00 | Cystitis |
| K150.00 | Acute cystitis |
| K15z.00 | Cystitis NOS |
| K190.00 | Urinary tract infection, site not specified |
| K190.11 | Recurrent urinary tract infection |
| K190100 | Pyuria, site not specified |
| K190200 | Postoperative urinary tract infection |
| K190300 | Recurrent urinary tract infection |
| K190311 | Recurrent UTI |
| K190400 | Chronic urinary tract infection |
| K190500 | Urinary tract infection |
| K190z00 | Urinary tract infection, site not specified NOS |
| SP07700 | Infect + inflam react due pros dev, implt+graft in urinary… |
| SP07Q00 | Catheter associated urinary tract infection |
| SP07Q11 | CAUTI – catheter associated urinary tract infection |
| K15y.00 | Other specified cystitis |
| K152.00 | Other chronic cystitis |
| K152y00 | Chronic cystitis unspecified |
| K155.00 | Recurrent cystitis |
| K15yz00 | Other cystitis NOS |
| 1AZ6000 | Mild lower urinary tract symptoms |
| 1AZ6100 | Moderate lower urinary tract syptoms |
| 1AZ6.00 | Lower urinary tract symptoms |
| Kyu5100 | [X] Other cystitis |
| L1666 | Urinary tract infection following delivery |
| L1668 | Urinary tract infection complicating pregnancy |
| L177 | Infections of bladder in pregnancy |
| 46X0.00 | Urine nitrite positive |
| 46X2.00 | Urine dipstick for nitrite |
| 46U3.00 | Urine culture – E. coli |
| 46U..00 | Urine culture |
| 46U3.11 | Urine culture – Escherich.coli |
| 46U7.00 | Urine culture – Pseudomonas |
| 46U2.00 | Urine culture – mixed growth |
| 46U4.00 | Urine culture – Proteus |
| 46U8.00 | Urine culture – Bacteria OS |
| 46U6.00 | Urine culture – Staph. Albus |
| 46U5.00 | Urine culture – Str. faecalis |
| 4617 | MSU = abnormal |

**Table S4. Read codes for recurrent UTI**

| Read code | Description |
| --- | --- |
| 1AG..00 | Recurrent urinary tract infections |
| K190.11 | Recurrent urinary tract infection |
| K190300 | Recurrent urinary tract infection |
| K190311 | Recurrent UTI |
| K190400 | Chronic urinary tract infection |
| K152z00 | Other chronic cystitis NOS |
| K152.00 | Other chronic cystitis |
| K152y00 | Chronic cystitis unspecified |
| K155.00 | Recurrent cystitis |
| K100.00 | Chronic pyelonephritis |
| K100000 | Chronic pyelonephritis without medullary necrosis |
| K100100 | Chronic pyelonephritis with medullary necrosis |
| K100400 | Nonobstructive reflux-associated chronic pyelonephritis |
| K100500 | Chronic obstructive pyelonephritis |
| K100600 | Calculous pyelonephritis |
| K100z00 | Chronic pyelonephritis NOS |
| K100200 | Chronic pyelitis |
| K105.00 | Chronic infective interstitial nephritis |

**Table S5. Read codes for comorbidities**

| Comorbidity | Read codes | Excluded Read codes | Date | Notes |
| --- | --- | --- | --- | --- |
| **QOF Conditions** |  |  |  |  |
| Cancer | B0%, B1%, B2%, B3, B30%, B31%, B32%, B34%, B35%,B36, B3y, B3z, B4%, B5%, B6%, Byu0, Byu1%, Buy2%, Byu3%, Byu4, Byu40, Byu41, Byu5%, Byu6%, Byu7%, Byu8%, Byu9%, ByuA%,ByuB%, ByuC%, ByuD%, ByuE%, K1323, K01w1, 68W24, C184 | B677 | Earliest ever |  |
| Chronic kidney disease | 1Z12,1Z13, 1Z14, 1Z15, 1Z16, 1Z1B, 1Z1C, 1Z1D, 1Z1E, 1Z1F, 1Z1G, 1Z1H, 1Z1J, 1Z1K, 1Z1L, 1Z1T, 1Z1V, 1Z1W, 1Z1X, 1Z1Y,1Z1Z, 1Z1a, 1Z1b, 1Z1c, 1Z1d, 1Z1e, 1Z1f,K053, K054, K055 |  | Earliest ever |  |
| Diabetes (T1 & T2) | C10, C109J, C109K, C10C, C10D, C10E%, C10F%, C10G%, C10H%, C10M%,  C10N%, PKyP, C10P%, C10Q | C10F8, 21263, 212H | Earliest ever | Diabetes resolved codes excluded if appearing after the latest diagnostic code |
| Heart failure | G58%, G1yz1, 662f, 662g, 662h,662i, I50% Heart failure |  | Earliest ever |  |
| Hypertension | G2, G20%, G24%, G25%, G26, G28, G2y, G2z, Gyu2, Gyu20 | 21261, 212K | Earliest ever | Hypertension resolved excluded |
| Obesity | 22K5, 22K7, 22KC, 22KD, 22KE |  | Earliest ever |  |
| **Other conditions** |  |  |  |  |
| Urinary catheters | 8156%, 66K9%, 7B241, 7B2B1, 7B2B2, 7B2B5, 7B2B7, 7B2B8, 7B2By, 7B2C1, 7B2C2, 7B2C9, 81, 8B3v, 8D74, 8E98, SP031, SP033, ZV536, ZV53A, ZV53E, ZV658 |  | Any between 01/04/12 – 31/03/17 |  |
| Urinary incontinence | R0831, Kyu5A, 1A230, 16F0, 1593, 1A24, K198, 317A, R0832, 16F%, 7B421, 679H%, 3940, 1A23, 1A24, 1A26, 3940, 3941, 39H, 39H0, 679H, 7B312, 7B312, 7B338, 7B33C, 7B421, 8C14, 8D7, 8D71, 8D7Z, 8E97, 8E970, 8H7w, 8HR6, 8HTX, 9Nl8, K586, R083, R083z |  | Earliest ever |  |
| Faecal incontinence | 16F0, R0761, 773Dy, 16F%, 773D, 3930%, 19E3%, 3931, 3930, R076, R076z |  | Earliest ever |  |
| Structural abnormalities urinary tract | See structural abnormalities code list in table 2.4. below |  | Earliest ever | Any of these codes |

**Table S6. Read codes for structural abnormalities of the renal tract**

| Read code | Description |
| --- | --- |
| 124L | FH: Malignant neoplasm of urinary bladder |
| 12F1 | FH: Polycystic kidney |
| 14D6 | H/O: urethral stricture |
| 1AH0 | Incomplete emptying of bladder |
| 4G6 | O/E - ureteric calculus |
| 4G7 | O/E - urethral calculus |
| 7B | Transplantation of kidney |
| 7B0 | Autotransplant of kidney |
| 7B01 | Total nephrectomy |
| 7B013 | Heminephrectomy for horseshoe kidney |
| 7B015 | Transplant nephrectomy |
| 7B02 | Partial nephrectomy |
| 7B020 | Heminephrectomy for duplex kidney |
| 7B021 | Division of isthmus of horseshoe kidney |
| 7B033 | Rovsing’s operation for polycystic kidney |
| 7B04 | Open repair of kidney |
| 7B043 | Plication of kidney |
| 7B045 | Plication and pyeloplasty of kidney |
| 7B04y | Other specified open repair of kidney |
| 7B050 | Unspecified open removal of calculus from kidney |
| 7B051 | Nephrostomy |
| 7B052 | Closure of nephrostomy |
| 7B05y | Other specified incision of kidney |
| 7B06 | Other open operations on kidney |
| 7B06y | Other specified other open operation on kidney |
| 7B070 | Nephroscopy and ultrasound lithotripsy of renal calculus |
| 7B071 | Nephroscopy & electrohydraulic lithotripsy of renal calculus |
| 7B072 | Nephroscopy and laser lithotripsy of renal calculus |
| 7B08 | Other therapeutic endoscopic operations on kidney |
| 7B080 | Endoscopic extirpation of lesion of kidney |
| 7B082 | Endoscopic de-roofing of multiple cysts of kidney |
| 7B083 | Endoscopic cryoablation of lesion of kidney |
| 7B084 | Endos endolum balloon rupt sten pelviureteric junct kidney |
| 7B093 | Diag endosc retrograde exam kidney and biopsy lesion kidney |
| 7B0A | Percutaneous puncture of kidney |
| 7B0A4 | Percutaneous nephrostomy |
| 7B0A8 | Percutaneous radiofrequency ablation of lesion of kidney |
| 7B0Ay | Other specified percutaneous puncture of kidney |
| 7B0C | Other operations on kidney |
| 7B0C1 | Maintenance of nephrostomy tube |
| 7B0Cy | Other specified other operation on kidney |
| 7B0F | Interventions associated with transplantation of kidney |
| 7B0Fy | OS interventions associated with transplantation of kidney |
| 7B0y | Other specified operations on kidney |
| 7B11 | Urinary diversion |
| 7B111 | Unspecified other urinary intestinal diversion |
| 7B112 | Revision of urinary diversion |
| 7B11B | Insertion of subcutaneous urinary diversion stent |
| 7B11y | Other specified urinary diversion |
| 7B122 | Ureteric reimplantation after urinary diversion |
| 7B130 | Anastomosis of ureter to bladder |
| 7B131 | Boari flap anastomosis of ureter to bladder |
| 7B170 | Nephroscopic laser lithotripsy of ureteric calculus |
| 7B173 | Nephroscopic insertion of ureteric stent |
| 7B18 | Ureteroscopic operations for ureteric calculus |
| 7B180 | Ureteroscopic laser lithotripsy of ureteric calculus |
| 7B183 | Ureteroscopic insertion of ureteric stent |
| 7B184 | Ureteroscopic removal of ureteric stent |
| 7B192 | Cystoscopic extraction of ureteric calculus |
| 7B193 | Cystoscopic catheter drainage for ureteric calculus |
| 7B194 | Cystoscopic dilation of ureter for drainage of calculus |
| 7B19y | Other specified cystoscopic removal of ureteric calculus |
| 7B1A1 | Endoscopic insertion of ureteric stent |
| 7B1A2 | Endoscopic removal of ureteric stent |
| 7B1A4 | Endoscopic replacement of ureteric stent |
| 7B1E5 | Percutaneous removal of ureteric stent |
| 7B1F% | Therapeutic endoscopic operations on urinary diversion |
| 7B1F0 | Endoscopic extraction of calculus of urinary diversion |
| 7B20 | Total excision of bladder |
| 7B20y | Other specified total excision of bladder |
| 7B21 | Partial excision of bladder |
| 7B210 | Diverticulectomy of bladder |
| 7B21y | Other specified partial excision of bladder |
| 7B22 | Enlargement or replacement of bladder |
| 7B227 | Ileal augmentation of bladder |
| 7B22y | Other specified enlargement of bladder |
| 7B23 | Other repair of bladder |
| 7B234 | Closure of exstrophy of bladder |
| 7B23y | Other specified other repair of bladder |
| 7B24 | Open drainage of bladder |
| 7B241 | Cystostomy and insertion of suprapubic catheter |
| 7B24y | Other specified open drainage of bladder |
| 7B25 | Open operations on contents of bladder |
| 7B26 | Other open operations on bladder |
| 7B262 | Open transection of bladder |
| 7B26y | Other specified other open operation on bladder |
| 7B274 | Rigid cystoscopic diathermy of lesion of bladder |
| 7B282 | Other endoscopic overdistension of bladder |
| 7B284 | Cystoscopic hydrostatic distension of bladder |
| 7B286 | Endoscopic hydrostatic distension of bladder |
| 7B29 | Other therapeutic cystoscopy |
| 7B293 | Endoscopic removal of blood clot from bladder |
| 7B2B0 | Urethral irrigation of bladder |
| 7B2C | Other operations on bladder |
| 7B2Cy | Other specified other operation on bladder |
| 7B2D | Operations on bladder |
| 7B2D0 | Suprapubic aspiration of bladder |
| 7B2Dy | Other specified operations on bladder |
| 7B2y | Other specified operations on bladder |
| 7B30 | Combin abdominal & vaginal ops support outlet female bladder |
| 7B300 | Abdominoperineal suspension of urethra |
| 7B31 | Abdominal operations to support outlet of female bladder |
| 7B311 | Retropubic suspension of bladder neck |
| 7B32 | Vaginal operations to support outlet of female bladder |
| 7B320 | Vaginal buttressing of urethra |
| 7B33 | Other open operations on outlet of female bladder |
| 7B334 | Insertion of sphincter around female bladder neck |
| 7B33y | Other specified open operation on outlet of female bladder |
| 7B34 | Therapeutic endoscopic operations outlet of female bladder |
| 7B343 | Endoscopic uroplastic injection outlet of female bladder |
| 7B35 | Other operations on outlet of female bladder |
| 7B351 | Dilation of outlet of female bladder |
| 7B35y | Other specified other operation on outlet of female bladder |
| 7B38 | Other open operations on outlet of male bladder |
| 7B38y | Other specified open operation on outlet of male bladder |
| 7B39 | Endoscopic resection of outlet of male bladder or prostate |
| 7B3A | Other therapeutic endoscopy on outlet of male bladder |
| 7B3A0 | Endoscopic external sphincterotomy of male bladder |
| 7B3A3 | Endoscopic uroplastic injection outlet of male bladder |
| 7B3B5 | Endoscopic insertion of prostatic stent |
| 7B3B7 | Endoscopic removal of prostatic stent |
| 7B3B8 | Endoscopic change of prostatic stent |
| 7B3C | Other operations on prostate or male bladder outlet |
| 7B3D0 | Endoscopic insertion of prostatic stent |
| 7B3D1 | Endoscopic removal of prostatic stent |
| 7B3F | Open operations on outlet of male bladder |
| 7B40 | Excision of urethra |
| 7B40y | Other specified excision of urethra |
| 7B41 | Repair of urethra |
| 7B410 | Other hypospadias repair |
| 7B413 | Unspecified reconstruction of urethra |
| 7B414 | Pull through of urethra |
| 7B41y | Other specified repair of urethra |
| 7B42 | Other open operations on urethra |
| 7B421 | Insertion of bulbar urethral prosthesis |
| 7B424 | Open extraction of calculus from urethra |
| 7B42y | Other specified open operation on urethra |
| 7B43 | Therapeutic endoscopy of urethra |
| 7B433 | Endoscopic urethral dilatation |
| 7B434 | Endoscopic insertion of urethral stent |
| 7B436 | Endoscopic removal of urethral stent |
| 7B43y | Other specified therapeutic endoscopy of urethra |
| 7B440 | Diagnostic urethroscopy and biopsy |
| 7B45 | Other operations on urethra |
| 7B452 | Calibration of urethra |
| 7B455 | Balloon dilatation of urethra |
| 7B45y | Other specified other operation on urethra |
| 7B463 | Dilatation of urethral meatus |
| 7B48% | Diagnostic endoscopic examination of urinary diversion |
| 7By | Other specified transplantation of kidney |
| 7L1D0 | Donation of kidney |
| B49 | Malignant neoplasm of urinary bladder |
| B490 | Malignant neoplasm of trigone of urinary bladder |
| B491 | Malignant neoplasm of dome of urinary bladder |
| B492 | Malignant neoplasm of lateral wall of urinary bladder |
| B493 | Malignant neoplasm of anterior wall of urinary bladder |
| B494 | Malignant neoplasm of posterior wall of urinary bladder |
| B498 | Local recurrence of malignant tumour of urinary bladder |
| B49y | Malignant neoplasm of other site of urinary bladder |
| B49y0 | Malignant neoplasm, overlapping lesion of bladder |
| B4A3 | Malignant neoplasm of urethra |
| B580 | Secondary malignant neoplasm of kidney |
| B5811 | Secondary malignant neoplasm of bladder |
| B5812 | Secondary malignant neoplasm of urethra |
| B7D0 | Benign neoplasm of renal parenchyma |
| B7D3 | Benign neoplasm of bladder |
| B7Dy0 | Benign neoplasm of urethra |
| B917 | Neoplasm of uncertain behaviour of bladder |
| B91z1 | Neoplasm of uncertain behaviour of kidney |
| BA04 | Neoplasm of unspecified nature of bladder |
| BBLJ | [M]Clear cell sarcoma of kidney |
| F2461 | Cauda equina syndrome with cord bladder |
| K070 | Atrophy of kidney |
| K09 | Atrophy of kidney |
| K090 | Unilateral small kidney |
| K113 | Hydronephrosis with ureteropelvic junction obstruction |
| K120 | Calculus of kidney |
| K130 | Nephroptosis |
| K131 | Hypertrophy of kidney |
| K1310 | Compensatory hypertrophy of single kidney |
| K132 | Acquired cyst of kidney |
| K134 | Other ureteric obstruction |
| K137 | Vesicoureteric reflux |
| K138 | Vascular disorders of kidney |
| K13y1 | Adhesions of kidney |
| K13z0 | Non-functioning kidney |
| K14 | Lower urinary tract calculus |
| K1400 | Calculus in diverticulum of bladder |
| K1401 | Other calculus in bladder |
| K141 | Calculus in urethra |
| K1511 | Panmural fibrosis of bladder |
| K15y3 | Malakoplakia of bladder |
| K16 | Other disorders of bladder |
| K160 | Bladder neck obstruction |
| K163 | Diverticulum of bladder |
| K164 | Atony of bladder |
| K165 | Other functional disorder of bladder |
| K1652 | Bladder outflow obstruction |
| K1654 | Unstable bladder |
| K168 | Amyloid of bladder |
| K16V0 | Neuropathic bladder |
| K16V1 | Overactive bladder |
| K16y0 | Calcified bladder |
| K16y1 | Contracted bladder |
| K16y4 | Irritable bladder |
| K16y5 | Trabeculation of bladder |
| K16y7 | Squamous metaplasia of bladder |
| K16y8 | Functional disorder of bladder |
| K18 | Urethral stricture |
| K180 | Infective urethral stricture |
| K181 | Traumatic urethral stricture |
| K182 | Postoperative urethral stricture |
| K18y | Other urethral stricture |
| K50y0 | Endometriosis of the bladder |
| Kyu52 | [X]Other neuromuscular dysfunction of bladder |
| Kyu53 | [X]Other specified disorders of bladder |
| Kyu56 | [X]Other urethral stricture |
| Kyu57 | [X]Other specified disorders of urethra |
| PD02 | Congenital absence of kidney |
| PD021 | Unilateral congenital absence of kidney |
| PD03 | Hypoplasia of kidney |
| PD04 | Dysplasia of kidney |
| PD1 | Congenital cystic kidney disease |
| PD11 | Polycystic kidney disease |
| PD110 | Polycystic kidneys, infantile type |
| PD111 | Polycystic kidneys, adult type |
| PD121 | Medullary cystic disease, adult type |
| PD13 | Multicystic renal dysplasia |
| PD30 | Accessory kidney |
| PD31 | Congenital calculus of kidney |
| PD32 | Congenital displaced kidney |
| PD33 | Discoid kidney |
| PD35 | Ectopic kidney |
| PD37 | Giant kidney |
| PD38 | Horseshoe kidney |
| PD39 | Hyperplasia of kidney |
| PD3A | Lobulation of kidney |
| PD3B | Malrotation of kidney |
| PD3D | Enlarged kidney |
| PD3E | Cake kidney |
| PD3F | Bifid kidney |
| PD47 | Congenital vesico-uretero-renal reflux |
| PD5 | Exstrophy of urinary bladder |
| PD50 | Ectopic bladder |
| PD60 | Congenital bladder neck obstruction |
| PD61 | Congenital obstruction of urethra |
| PD610 | Atresia of anterior urethra |
| PD611 | Stenosis of anterior urethra |
| PD62 | Congenital urethral valvular stricture |
| PD63 | Congenital urinary meatus stricture |
| PD64 | Congenital vesicourethral orifice stricture |
| PD66 | Impervious urethra |
| PD8 | Congenital abnormality of the kidney |
| PD80 | Duplex kidney |
| PDy0 | Congenital absence of bladder |
| PDy2 | Accessory bladder |
| PDy3 | Accessory urethra |
| PDy8 | Congenital prolapse of urethra |
| PDy9 | Double urethra |
| PDz0 | Unspecified anomaly of kidney |
| PDz2 | Unspecified anomaly of bladder |
| PDz3 | Unspecified anomaly of urethra |
| Pyu73 | [X]Other specified congenital malformations of kidney |
| Pyu75 | [X]Other congenital malformations of bladder and urethra |
| S76 | Injury to kidney |
| S760 | Closed injury of kidney |
| S7700 | Closed injury of bladder |
| S7701 | Closed injury of urethra |
| S7710 | Open injury of bladder |
| S7711 | Open injury of urethra |
| S777 | Injury of bladder |
| S778 | Injury of urethra |
| SP038 | Blocked ureteric stent |
| SP08P | Stenosis of vein of transplanted kidney |
| ZV105 | [V]Personal history of malignant neoplasm of urinary organ |
| ZV130 | [V]Personal history of urinary system disorder |
| ZV446 | [V]Has other artificial opening of urinary tract |
| ZV556 | [V]Attention to other artificial opening of urinary tract |
| ZV6G5 | [V]Acquired absence of kidney |

**Table S7. Devices and products used to identify urinary catheters, urinary incontinence and faecal incontinence**

| Product prescribed | BNF chapter | Additional text search terms |
| --- | --- | --- |
| Catheters prescribed | 21.2 Catheters | Astra Tech Lofric Hydro-Kit II Nelaton catheter, container + bag female 12ch, 9851200  Astra Tech Lofric Hydro-Kit II Nelaton catheter, container + bag male 14ch, 9831400  Astra Tech Lofric Nelaton Catheter, female 10 ch, 941000  Astra Tech Lofric Nelaton Catheter, female 15 cm 10 ch, 981000  Astra Tech Lofric Nelaton Catheter, male 16 ch, 901600  Astra Tech Lofric Primo Nelaton Catheter (female 20cm) 16 ch, 4131625  Astra Tech Lofric Primo Nelaton catheter (male) 14ch, 9601400  Astra Tech Lofric Primo Nelaton catheter (male) 16ch, 9601600 Bard Uriplan 10 Cm Inlet Tube Leg Bag 500 ml, d5m  Coloplast Simpla Catheter Valve T180 LoFric catheter female 12Ch 4031225 20cm (Astra Tech Ltd)  LoFric catheter male 14Ch 901400 (Astra Tech Ltd)  Lofric Nelaton Catheter, Male 16 ch, 901600  LoFric Primo catheter male 16Ch 9601600 (Astra Tech Ltd)  Uriplan sterile leg bag D5M 500ml bag, 10cm inlet tube (Bard Ltd) |
| Catheter maintenance products | 21.13 Catheter maintenance solutions | Optiflo G Catheter Solution Citric Acid (Suby G) 3.23%, 50ml  Optiflo S Catheter Solution Saline 0.9 %, 100 ml  Sodium Chloride Bladder Irrigation 0.9 %, 1 litre  Solution G Catheter Maintenance Solution 50 ml sachet |
| Incontinence Products | 22. Incontinence appliances | Attends Incontinence Pads Normal +, 60cm x 60cm |
| Suprapubic Appliances | 21.5. Suprapubic appliances |  |
| Urostomy bags | 23.96. Urostomy bags |  |
| Urostomy sets | 23.98. Urostomy sets |  |
| Urostomy bags | 23.96. Urostomy bags |  |

**Table S8. ICD-10 codes identifying urinary infection-related hospital admission**

|  | ICD-10 code | Description |
| --- | --- | --- |
| **Lower** **UTI** | N30.0 | Acute cystitis |
|  | N30.9 | Cystitis, unspecified |
|  | N30.8 | Other cystitis |
|  | N39.0 | Urinary tract infection, site not specified |
| **Upper** **UTI** | N10 | Acute tubulo-interstitial nephritis |
|  | N12 | Tubulo-interstitial nephritis, not specified as acute or chronic |
|  | N13.6 | Pyonephrosis |
|  | N15.1 | Renal and perinephric abscess |
|  | N15.8 | Other specified renal tubulo-interstitial diseases |
|  | N15.9 | Renal tubulo-interstitial disease, unspecified |
|  | N16.0 | Renal tubulo-interstitial disorders in infectious and parasitic diseases classified elsewhere |
|  | N28.8 | Other specified disorders of kidney and ureter |
|  | N34.0 | Urethral abscess |
|  | N34.1 | Nonspecific urethritis |
|  | N34.2 | Other urethritis |
|  | N34.3 | Urethral syndrome, unspecified |
|  | N41.0 | Acute prostatitis |
|  | N41.1 | Chronic prostatitis |
|  | N41.2 | Abscess of prostate |
|  | N41.3 | Prostatocystitis |
|  | N41.8 | Other inflammatory diseases of prostate |
|  | N41.9 | Inflammatory disease of prostate, unspecified |
|  | N11.0 | Nonobstructive reflux-associated chronic pyelonephritis |
|  | N11.1 | Chronic obstructive pyelonephritis |
|  | N11.8 | Other chronic tubulo-interstitial nephritis |
|  | N11.9 | Chronic tubulo-interstitial nephritis, unspecified |
| **Sepsis/other** | R57.2 | Septic shock |
|  | R65.1 | Systemic Inflammatory Response Syndrome of infectious origin with organ failure |
|  | R65.0 | Systemic Inflammatory Response Syndrome of infectious origin without organ failure |
|  | A40.1 | Sepsis due to streptococcus, group B |
|  | A41.5 | Sepsis due to other Gram-negative organisms |
|  | A41.8 | Other specified sepsis |
|  | A41.9 | Sepsis, unspecified |
|  | A49.9 | Bacterial infection, unspecified - Bacteraemia NOS |
|  | B96.1 | Klebsiella pneumoniae [K. pneumoniae] as the cause for diseases classified to other chapters |
|  | B96.2 | Escherichia coli [E. coli] as the cause for diseases classified to other chapters |
|  | B96.4 | Proteus (mirabilis) (morganii) as the cause for diseases classified to other chapters |
|  | B96.5 | Pseudomonas (aeruginosa) as the cause for diseases classified to other chapters |
|  | R65.0 | Severe sepsis without septic shock |
|  | R65.1 | Severe sepsis with septic shock |
